# Surgery for Oligometastatic Pancreatic Cancer: Defining Biologic Resectability

**DOI:** 10.1245/s10434-024-15129-8

**Published:** 2024-03-19

**Authors:** Shruti Koti, Lyudmyla Demyan, Gary Deutsch, Matthew Weiss

**Affiliations:** 1 Department of General Surgery, Northwell Health, Queens, NY USA; 2 Northwell Health Cancer Institute, Northwell Health, New Hyde Park, NY USA; 3 Donald and Barbara Zucker School of Medicine at Hofstra/Northwell, Hempstead, NY USA

**Keywords:** Pancreatic cancer, Metastasectomy, Oigometastatic PDAC, Biologic resectability, Biomarkers, Oncologic outcomes

## Abstract

Pancreatic ductal adenocarcinoma (PDAC) is most often metastatic at diagnosis. As systemic therapy continues to improve alongside advanced surgical techniques, the focus has shifted toward defining biologic, rather than technical, resectability. Several centers have reported metastasectomy for oligometastatic PDAC, yet the indications and potential benefits remain unclear. In this review, we attempt to define oligometastatic disease in PDAC and to explore the rationale for metastasectomy. We evaluate the existing evidence for metastasectomy in liver, peritoneum, and lung individually, assessing the safety and oncologic outcomes for each. Furthermore, we explore contemporary biomarkers of biological resectability in oligometastatic PDAC, including radiographic findings, biochemical markers (such as CA 19-9 and CEA), inflammatory markers (including neutrophil-to-lymphocyte ratio, C-reactive protein, and scoring indices), and liquid biopsy techniques. With careful consideration of existing data, we explore the concept of biologic resectability in guiding patient selection for metastasectomy in PDAC.

Pancreatic ductal adenocarcinoma (PDAC) is an aggressive malignancy, which by 2030 will be the second-leading cause of cancer-related deaths in the United States.^1^ Despite advances in systemic therapy and surgical techniques, most patients present with unresectable metastatic disease. Twenty-five percent of patients are considered “borderline resectable” or “locally advanced” as defined by National Comprehensive Cancer Network (NCCN) and National Cancer Institute (NCI) treatment guidelines.^2^

In recent years, advances in the combination therapies FOLFIRINOX and gemcitabine-based perioperative treatment have increased overall survival (OS) in the metastatic setting, and these trials have impacted the treatment paradigm in borderline resectable disease.^3–5^ Increased utilization of multiagent chemotherapy has opened up possibilities for surgical intervention in cases that were once thought to be unresectable. In many cases, the discussion has shifted from one of “technical” resectability to those of “biologic” resectability.^6,7^

Growing literature suggests that clinicopathologic risk factors and tumor biology may impact the pattern of disease and site of the first recurrence. Sites of recurrence after curative-intent resection include liver (25.6%), locoregional (20.8%), peritoneal dissemination (13.5%), and lung (11.4%); additionally, many cases may present with multisite recurrence.^8^ Studies comparing site of metastatic disease have demonstrated differences that suggest multiple PDAC phenotypes, each associated with a different type of tumor biology and behavior.^9^

While historically, the presence of metastases in PDAC has been a contraindication for curative-intent resection, population and observational studies in the past decade have reported technical feasibility and safety of metastasectomy for oligometastatic disease. Metastasectomy has already been well-documented in colon cancer, melanoma, renal cell carcinoma, and certain types of sarcoma.^10–13^ Although there are several studies on metastasectomy in PDAC, its adoption is still controversial, and evidence is not yet strong enough to influence any guidelines. The wide variation in tumor biology, response to therapy, and recurrence raises the question: Are there certain biologic and patient-specific factors that may dictate oncologic success of metastasectomy?

In this paper, we will attempt to define oligometastatic disease in PDAC, review recent literature on metastasectomy to clarify safety and oncologic outcomes, and finally, highlight the nuances of patient and tumor characteristics to explore the potential role for surgical resection of oligometastatic pancreatic cancer.

## Defining Oligometastatic Disease

The term “oligometastasis” was first described in 1995 by Hellman and Weichselbaum, who theorized that early in the progression of a malignancy, a limited number of metastases may appear, before to the development of exponential metastatic growth.^14^ Since then, several others have expanded upon or differed from this hypothesis. A study by Lussier et al. comparing microRNA expression of tumor samples from oligometastatic patients found that some of these patients failed to progress to polymetastases; these samples were characterized by distinct microRNA features.^15^ In colorectal cancer, several studies have identified specific gene mutations, such as ERBB2, and regression of key-driver gene mutations (KRAS, PIK3CA) that are associated with oligometastatic clinical behavior.^11,16,17^ These findings suggest that oligometastatic disease may be a distinct entity from polymetastatic disease, rather than an earlier timepoint in the inevitable timeline of metastatic spread.

Use of the term “oligometastasis” has increased in recent years; however, its definitions and implications have remained nebulous. A recent metanalysis by Rim et al. investigating the role of local consolidative therapy for oligometastasis found that 48.1% of studies defined oligometastasis as up to five lesions, 7.4% up to four lesions, and 25.9% as up to three lesions.^18^ These cutoffs are seemingly arbitrary, and this lack of definition also is reflected in clinical practice. A survey of medical and radiation oncologists found no common understanding of oligometastatic disease and significant variability in treatment recommendations.^19^

A recent ASTRO/ESTRO (American Society for Radiation Oncology/European Society for Radiotherapy and Oncology) consensus study proposed a definition for oligometastatic disease that centered on the ability to deliver safe and meaningful radiotherapy with curative intent to all metastatic sites.^20^ Perhaps, then, the surgeon should define oligometastatic disease as that which can be safely operated on with a reasonable chance of cure or significant prolongation of life. The reality is that the clinical state of “oligometastasis” is poorly understood. To date, the decision of whether to operate on these patients is limited by surgeon and institute experience and individual patient preference.

## Rationale for Metastasectomy in Pancreatic Ductal Adenocarcinoma

Metastasectomy may be justified when it is safe and offers a survival benefit, improved quality of life, or a possibility of cure. Metastasectomy for patients with stage IV colon cancer now offers a potential for cure, and liver metastasectomy in carefully selected patients provides a 40–60% chance of 5 year survival.^16,21^ In patients with pancreatic neuroendocrine tumors (PNETs) undergoing liver-directed therapy, the 5 year overall survival has been reported as up to 80%.^22^ In the case of metastatic melanoma, BRAF inhibitors, and CTLA-4 and PD-1 inhibitors have been shown to induce a rapid response and conversion to oligometastatic disease, creating the opportunity for metastasectomy and resulting in 40% 5 year survival in patients with stage IV disease.^23,24^ These studies demonstrate the importance of systemic control to allow for metastasectomy.

It has been observed that there are subtypes of metastatic PDAC that may respond differently to the same treatment. This is in part because of complex genomic rearrangements and nonconventional mutagenesis of key driver genes (KRAS, TP53, SMAD4, and CDKN2A) resulting in rapid tumor progression.^25^ These genetic and molecular differences may lead to differences in tissue tropism, and subsequently, differences in response to therapy and patient outcomes. In the following sections, we will separately approach liver, lung, and peritoneum as sites of metastatic PDAC, and review the existing data surrounding metastasectomy.

## Liver Metastases in PDAC

The liver is the most common organ for initial metastatic spread or distant recurrence in patients with pancreatic cancer.^8^ Of patients presenting with metastatic PDAC, 87.7% have synchronous liver metastases. Additionally, despite advances in imaging technology, 8% of patients may have occult metastatic disease at the time of surgical exploration.^26^ Furthermore, in patients with limited local PDAC who have undergone resection, 26.5% later develop metachronous liver disease.^27^

This liver tropism may be explained by the portal venous blood supply and lymphatic drainage, which provide means for hematogenous and lymphatic spread, respectively. There also is evidence that genetic alterations in TP53 and TGF-beta signaling might predict the pattern of metastatic progression.^28^ Interestingly, after metastasectomy, the recurrence of metastatic disease occurs in the same organ in most cases, supporting the concept of molecular programing for metastatic disease.^29^

### Role of Preoperative Chemotherapy in Downstaging PDAC Liver Metastases

Systemic therapies, such as FOLFIRINOX and gemcitabine + nab-paclitaxel, have improved clinical outcomes and survival in patients with metastatic PDAC (mPDAC).^30,31^ Frigerio and colleagues performed a prospective study of mPDAC patients, in which 24 patients with mPDAC had complete radiologic disappearance of liver metastasis after preoperative chemotherapy and underwent curative-intent resection of the primary tumor. Eighty-eight percent of these patients had an R0 resection of the primary tumor, and the median disease-free survival was 21 months after diagnosis.^32^ Despite the small sample size, this study shows the potential value of preoperative chemotherapy in controlling metastatic disease and rendering the tumor operable.

Nagai et al. investigated PDAC patients undergoing liver resection for isolated metastasis to identify favorable factors associated with survival. The overall survival in patients who received preoperative chemotherapy followed by surgery was 24 months from surgery compared with only 10.6 months in patients who underwent just “upfront” surgery (*p* = 0.01).^33^ These findings demonstrate the importance of systemic treatment in conjunction with operative intervention to help to identify patients with more favorable tumor biology.

Despite several studies evaluating liver metastases, the median survival for patients with liver metastases from PDAC has remained poor compared with other sites of metastases, regardless of treatment approach.^34^ In a study by Groot et al. reviewing PDAC patients who experienced recurrence after pancreatectomy, patients with multiple-site recurrence (4.7 months) or liver-only recurrence (7.2 months) had significantly worse median survival compared with lung-only recurrence (15.5 months) or local-only recurrence (9.7 months).^35^ In the absence of precise predictors of biologic behavior, all patients with metastatic PDAC should be evaluated for receipt of systemic therapy before consideration of liver resection.

### Surgical Outcomes for Synchronous and Metachronous Liver Resections

During the past decade, several studies have published surgical outcomes for patients undergoing liver resection for synchronous or metachronous metastatic lesions in PDAC (Table 1). Bachellier et al. reviewed 92 patients who underwent resection of PDAC with synchronous liver metastases.^36^ A variety of operations were undertaken, including pancreaticoduodenectomy (53.2%), total pancreatectomy (5.4%), and distal pancreatectomy with splenectomy (41.3%). Venous and arterial resections were performed in 4% and 8% of patients, respectively, and 18.4% of patients had associated visceral resections (stomach and left colon). With regards to liver metastases, 50% of patients had only a single liver lesion, and liver resections were mostly nonanatomic or minor resections (67%), RFA only (20.6%), or resection and RFA (11.9%), with one patient undergoing left hepatectomy. The overall 90 day morbidity and mortality rates in the entire cohort were 40.2% and 5.4%, respectively. The only difference in postoperative outcomes was higher postoperative pancreatic fistula in left pancreatic resection versus right pancreatic resection (18% vs. 0%). Given the variability in operative approach and differences in extent of resection seen across publications (Table 1), it is difficult to establish broad guidelines for liver resection in PDAC with respect to surgical outcomes.

**Table 1:** Surgical outcomes in patients undergoing synchronous liver metastasectomy for pancreatic ductal adenocarcinoma

First author (year)	No. patients	Median liver mets	Operation for primary lesion	Operation for liver lesion	Postoperative morbidity (%)	Reoperation (%)	30 day postoperative mortality (%)
Nagai^33^ (2023)	47	NR	PD 57% DP 43%	NR	45	NR	0
Bachellier^36^ (2022)	92	3	PD 53% DP 41% TP 6%	Resection 67% RFA 21% Resection + RFA 12%	40	NR	5.4
Safi^37^ (2021)	35	1 (1–4)	NR	Atypical resection 100%	NR	NR	7.9
Shao^38^ (2021)	50	NR	NR	Resection 90% Resection + RFA 10%	NR	2	NR
Yang^39^ (2019)	48	NR	PD 41.6% DP 58.3%	Wedge 89.5% Other 10.3%	14.5	0	4
Andreou^40^ (2018)	76	1	PD 67% DP 25% TP 8%	Major resection 8% Atypical resection 92%	50	12	5
Hackert^41^ (2017)	62	1–3	PD 43% DP 41% TP 17%	Major resection 14% Atypical resection 86%	45	3.2	1.6
Tachezy^42^ (2016)	69	2	PD 60% DP 36% TP 3%	Median of 2 liver resections (range 1–11)	68	6	1
Shi^43^ (2016)	30	NR	PD 37% DP 60% TP 3%	NR	73	NR	0

Studies with liver-only synchronous metastases with sample size *n* ≥ 30 from 2010 to 2023 are included

*NR* not reported, *PD* pancreaticoduodenectomy, *DP* distal pancreatectomy, *TP* total pancreatectomy, *RFA* radiofrequency ablation

There also have been studies examining the management of metachronous liver lesions from PDAC. In a retrospective review of 128 patients, Hackert et al. evaluated patients undergoing primary tumor and metastasis resection for PDAC and found a postoperative morbidity and mortality of 45% and 2.9%, respectively, for patients undergoing synchronous resection, and for patients undergoing liver resection only for metachronous metastases, it was 21.7% and 4.3%, respectively.^41^ The majority of patients with liver metastases (86%) had nonanatomic resections of one to four lesions. Only 14% of patients received formal resections, including bisegmentectomies and right/extended right hepatectomies.

Another multicenter study by Schwarz et al. identified patients who underwent hepatectomy for metachronous PDAC liver metastases to assess postoperative outcomes and overall survival.^44^ The median number of metastases in the group was 1, and overall postoperative morbidity was found to be 32%, which is comparable to existing data regarding postoperative morbidity after liver resection.

The majority of these studies have been performed in experienced centers by surgeons specializing in hepatobiliary operations. Although these data show acceptable postoperative morbidity and mortality, the feasibility and safety of liver resection for PDAC metastases should be evaluated in the context of metastatic tumor burden and surgeon and center experience.

### Oncological Outcomes in Patients Undergoing Resection for Synchronous Liver Metastases

Current studies on liver metastasectomy in PDAC have reported a variety of oncologic outcomes, although sample sizes are small, and there are discrepancies in reported outcome variables (Table 2).

**Table 2:** Oncologic outcomes in patients undergoing synchronous liver metastasectomy for pancreatic ductal adenocarcinoma

First author (year)	No. patients	Median age	% Receiving preoperative chemotherapy (%)	% Receiving adjuvant therapy (%)	R0 at pancreatic resection (%)	OS (months)	DFS (months)
Nagai^33^ (2023)	47	62	68	60	81	21.9	6.1
Bachellier^36^ (2022)	92	63.5	56.5	85	NR	18	5.4
Safi^37^ (2021)	35	67	11.4	60	48.6	10.3	NR
Shao^38^ (2021)	50	63	82	76	46	16	NR
Yang^39^ (2019)	48	62	25	79	100	7.8	NR
Andreou^40^ (2018)	76	64	5	72	82	NR	NR
Hackert^41^ (2017)	62	60.4	32.2	74.2	18.8	10.6	NR
Shi^43^ (2016)	30	62.2 (mean)	NR	NR	NR	15.7	NR
Tachezy^42^ (2016)	69	65	14	80	58	14.5	NR

Studies with liver-only synchronous metastases with *n* ≥ 30 from 2010 to 2023 are included

*OS* overall survival, *DFS* disease-free survival, *NR* not reported

In a small case-control study, Kandel et al. reported a median OS of 2.7 years in patients (*n* = 6) who underwent preoperative chemotherapy and synchronous hepatic resection, R0/R1 primary tumor resection, and adjuvant therapy. ^45^ The median OS in this cohort was similar that of PDAC patients without metastatic disease (*n* = 8) who underwent curative intent resection ± preoperative chemotherapy (OS 2.02 years) and was superior to the median OS (0.98 years) in patients with metastatic disease (*n* = 18), who received chemotherapy only. Shao et al. found that overall survival in patients who had curative-intent resection with liver metastasectomy had an improved survival (16 months) compared with a matched control group of patients who had palliative surgery only (6 months).^38^

As mentioned previously, Hackert and colleagues presented one of the largest series on liver metastasectomy (*n* = 128) in patients with isolated liver oligometastatic disease (with 1–3 liver metastases). However, only 20 patients (15.6%) received preoperative chemotherapy, and only 57% received postoperative adjuvant chemotherapy (gemcitabine being the most commonly administered, 79.5%). Patients with synchronous resection had median survival of 10.6 months, and metachronous resection had a median survival of 14.8 months from liver resection.^41^

Frigerio et al. performed a retrospective analysis of 52 patients with liver-only synchronous metastasis, with 73.1% of patients having greater than two liver metastases.^46^ All patients received preoperative chemotherapy (63.5% FOLFIRINOX, 36.5% gemcitabine-based) and had complete regression of metastatic lesions before surgical intervention on cross-sectional imaging. Of total patients, 67.3% had normalized Ca19-9 posttreatment. With an 86.5% R0 resection rate, the overall survival from diagnosis was 37.2 months, and median disease-free survival (DFS) after pancreatectomy was 16.5 months. Of total patients, 75% experienced recurrence, and multivariate analysis found omission of adjuvant therapy to be associated with recurrence. The improved overall survival in this series demonstrates the importance of chemotherapy to help select for favorable biology.

Some of these studies also have sought to identify factors associated with improved prognosis in attempts to guide patient selection. Frigerio et al. found that neutrophil-to-lymphocyte ratio <1.7 was significantly associated with improved overall survival and disease-free survival.^46^ Bachellier et al. identified that Ca19-9 <500 at diagnosis, R0 resection, and administration of adjuvant chemotherapy were independent prognostic factors for overall survival.^36^ These factors are further discussed in subsequent sections.

There are two ongoing clinical trials that will prospectively evaluate the potential benefit of surgery and the role of perioperative chemotherapy for oligometastatic PDAC to the liver. The first is the CSPAC-1 trial, opened in 2018 by the Chinese Study Group for Pancreatic Cancer.^47^ This is a phase 3 trial that will include 1000–1200 patients who meet inclusion criteria of: three or fewer lesions anywhere in the liver; a pathologic diagnosis of PDAC; and ECOG 0/1. After first-line chemotherapy, response will be assessed via RECIST criteria, and patients entering the second step of the trial will be randomized to simultaneous resection of primary pancreatic cancer and liver metastases, or standard chemotherapy. The primary endpoint is overall survival from the time of enrolment.

The second clinical trial, HOLIPANC, opened in Germany in 2021, is a single-arm phase 2 trial in which data will be collected from patients with oligometastatic PDAC getting chemotherapy with the NAPOX (liposomal irinotecan, oxaliplatin, 5-fluouracil, folinic acid) chemotherapy regimen, followed by R0/R1 resection.^48^ The results of these clinical trials help elucidate predictors of better biology and the potential role for resecting synchronous liver metastases.

### Oncological Outcomes in Patients Undergoing Resection for Metachronous Liver Metastases

Studies have reported favorable oncological outcomes in patients undergoing multimodal therapy for metachronous liver metastasis. Schwarz et al. performed a retrospective multicenter study of 25 patients undergoing hepatectomy for metachronous PDAC liver metastases and found that the median OS was 36.8 months from diagnosis compared with 9.2 months in patients who received chemotherapy only.^44^ A study by Zanini et al. found that median OS was significantly higher in patients with metachronous metastases undergoing liver resection compared with synchronous metastases.^49^ These studies suggest that surgical intervention for metachronous metastases may be associated with improved survival for certain patients.

In a retrospective study, Mitsuka et al. showed that patients with solitary metachronous liver metastases who underwent liver resection had improved median survival (55 months) compared with those patients who did not undergo liver resection (17.5 months).^50^ In this study, surgical resection was offered to patients who had no evidence of disease progression based on CT imaging during a 3 month observational period and were considered for a second metastasectomy if disease-free interval (DFI) was >12 months from the first liver resection. These data suggest that as in other malignancies, the DFI may be a good predictor of patients that could benefit from surgical resection of oligometastases.

## Peritoneal Metastases in PDAC

Peritoneal carcinomatosis (PC) is a hallmark of advanced-stage disease and historically has been associated with very poor outcomes, regardless of the primary tumor site. However, in patients with colorectal cancer (CRC), peritoneal carcinomatosis (PC) has been reclassified as locoregional disease, which drastically changed the surgical approach and allowed for use of cytoreductive surgery (CRS) with intraperitoneal chemotherapy (IPC).^51–53^

A prospective clinical trial by Yamada et al. investigated patients undergoing surgery for resectable PDAC, and if peritoneal dissemination or positive peritoneal cytology was encountered during staging surgery, intraperitoneal paclitaxel was administered.^54^ Of these 79 patients, 20.3% then underwent pancreatectomy for the primary tumor. However, after surgery, 75% of patients experienced recurrence. Such data suggest that in contrast to CRC, peritoneal carcinomatosis in PDAC may be far more aggressive and represent an advanced state of disease.

Despite these findings, there have been attempts at downstaging peritoneal disease in PDAC. Yamamoto and colleagues compared 43 patients receiving intraperitoneal chemotherapy (IPC) to 49 patients who received standard chemotherapy.^55^ The overall median survival time was longer in patients who underwent surgical resection than those who did not (27.4 months vs. 11.3 months). These data suggest that surgical interventions may provide meaningful extensions to overall survival after peritoneal metastases are downstaged via multimodal therapies.

Expanding on this principle, a newer technique, pressurized intraperitoneal aerosol chemotherapy (PIPAC) has been utilized in patients with peritoneal diseases mainly for palliation in patients with PC from various primary cancers.^56,57^ While several studies have demonstrated safety, ongoing prospective clinical trials are needed to evaluate efficacy.

## Lung Metastases in PDAC

The lung is a common site for metastases in patients with PDAC; however, unlike hepatic metastases, lung metastases are most frequently metachronous, presenting as a late recurrence of PDAC. Patients with pulmonary metastases have been shown to have a longer time between pancreatectomy and recurrence and also have better OS than those with other types of recurrence.^58,59^ Pulmonary metastasectomy (PM) has in recent years been recognized as a procedure for patients with PDAC with reported 5 year survival of 31.1–69.8%, although data are limited (Table 3).^60–63^

**Table 3:** Outcomes in patients undergoing surgery for metachronous pulmonary recurrence after resection of PDAC

First author (year)	No. patients	Median mets (range)	Median DFI after pancreatic resection (months)	Morbidity (%)	Postoperative mortality (%)	OS (months)	5 year OS (%)	DFS (months)
Homma^59^ (2022)	32	NR	27.8	NR	NR	NR	NR	25
Kaiho^62^ (2019)	12	1.5 (1–4)	41.1	NR	NR	NR	69.8	NR
Groot^58^ (2019)	19	1	24.3	15.7	0	27.1	NR	NR
Ilmer^63^ (2019)	15	NR	17	8.3	0	26	NR	18
Yasuwaka^60^ (2017)	12	1 (1–3)	32	0	0	47	31.2	NR
Robinson^64^ (2016)	16	NR	24	18.7	0	28	37.1	NR

Studies with lung-only metachronous metastases with *n*≥10 from 2010 to 2023 are included

*OS* overall survival, *DFI* disease-free interval, *DFS* disease-free survival, *NR* not reported

Several studies have evaluated oncologic outcomes in patients undergoing pulmonary metastasectomy. Yun et al. identified 83 patients in their study who, after pancreatectomy, developed metachronous pulmonary metastases (27.7% single metastasis, 34.9% oligometastases with 2–5 lesions, and 37.3% multiple metastases). In the entire study population, the 5 year OS was 60.6% in patients who underwent PM compared with 6.2% in patients who received only chemotherapy or supportive care; however, overall survival also was directly related to the number of metastases.^65^ These differences in the scale of improvement are likely explained by patient selection.

Several studies have proposed factors associated with improved survival after PM or lobectomy. Nakajima et al. evaluated multiple case reports and found that a disease-free interval (DFI) >20 months and size of lung metastases <1.6 cm were associated with longer survival after lobectomy.^66^ Other studies have investigated biologic factors. Homma et al. found that higher numbers of tumor-infiltrating lymphocytes in the lung metastases and CD8+ lymphocytes in the primary PDAC specimen were a favorable prognostic factor.^59^

At this time, cautiously proceeding with metastasectomy for metachronous pulmonary lesions from PDAC may be safe and efficacious in carefully selected patients. Futures studies are needed to better define a biologically distinct subgroup of patients in whom metastasectomy is indicated.

## Prognostic Factors for Improved Outcomes After Metastasectomy

### Radiographic Findings

Macroscopically, primary tumor size larger than 2–3 cm has been associated with worse outcomes in some studies, whereas R0 resection is a significant indicator of improved survival.^42,67,68^ More than five liver metastases also has found to be associated with worse survival.^69,70^ Reports on the significance of the location of liver metastasis (central vs. peripheral) and lung lesions are not conclusive, and the significance of these factors in predicting surgical and survival outcomes for patients with mPDAC is unknown.

Radiographic response, or tumor shrinkage, has been widely used as a surrogate of therapy response and tumor behavior, although there are currently no consensus guidelines on what should be used as a surgical indication for curative-intent resection. Frigerio and colleagues used the criteria of radiographic response in parallel with normalized CA 19-9 levels while receiving preoperative chemotherapy, although the majority of these patients recurred after surgery.^32^ A clinical response, as measured by Response Evaluation Criteria in Solid Tumors (RECIST), has been widely used in other tumors and is now gaining popularity in PDAC. In a review of 11 studies, Satoi et al. found that patients with unresectable PDAC with complete or partial response based on RECIST criteria, in parallel with CA 19-9 <150 U/ml following preoperative chemotherapy, had better OS after curative intent-resection than patients with stable disease.^71^

An alternative criterion, PET Response Criteria in Solid Tumors (PERCIST), also has been proposed.^72^ Additionally, Abdelrahaman et al. found that in patients with borderline or locally advanced PDAC undergoing preoperative chemotherapy followed by resection, metabolic response as measured by FDG-PET was the largest independent preoperative predictor of pathologic response, recurrence-free survival, and overall survival, even when taking into account biochemical markers, such as Ca19-9.^73,74^ Such radiographic criteria may be useful in determining response after initial downstaging treatment, although they have yet to be implemented in any prospective clinical trials.

### Systemic Biomarkers

Currently, the only FDA-approved PDAC biomarker is CA 19-9, with a median sensitivity of 79% and specificity of 82%. Takeda et al. identified that patients with liver-only metastases had a median Ca19-9 of 2780 U/mL compared with 6361 U/mL in patients with multiorgan metastases. On multivariate analysis, it was found that patients with oligometastatic liver disease with a Ca19-9 <1000 U/mL had improved overall survival. Additionally, 13 patients undergoing surgery after receipt of preoperative chemotherapy had an improved overall survival of 54.6 months compared with 20.8 months in patients who did not undergo surgery. While the range of pretreatment Ca19-9 values in the surgery group was wide, ranging from 4 to 50,000 U/mL, all patients in this group had normalization of Ca19-9 to 36 U/mL or less after preoperative chemotherapy.^75^

Tanaka et al. found that after FOLFIRINOX therapy, a CA 19-9 delta score (CA 19-9 postchemotherapy—CA 19-9 prechemotherapy) of 870 U/ml had 48% sensitivity and 81% specificity in predicting successful liver metastasectomy and primary tumor resection.^8^ These data suggest that CA19-9 reduction after preoperative chemotherapy may help to guide the decision to pursue metastasectomy.

While CA 19-9 has the potential to be used as marker of tumor response, variations in the data and a lack of clear cutoff values compromises its generalizability. In the study done by Frigerio at al., patients with synchronous liver metastases whose Ca19-9 had normalized after perioperative chemotherapy were selected for resection, and this was found to correlate significantly with improved OS.^32^ However, despite specifying a Ca19-9 decrease >50% relative to baseline as a selection criteria for metastasectomy, within this group, neither Ca19-9 decrease, nor posttreatment normalization were independently associated with survival.^46^ Additionally, and perhaps more importantly, approximately 5–7% of the population, belonging to the Le(a−b−) blood group, are unable to express Ca19-9, which compromises its ability to be used as a universal solitary biochemical marker.^76,77^

Carcinoembryonic antigen (CEA) also has been proposed as a biomarker. Studies in patients with pancreatic cancer have demonstrated that an elevated CEA at diagnosis was associated with a poorer overall survival compared with patients with a normal serum CEA.^78^ In a study by Hank et al., 93 patients with oligometastatic disease underwent metastasectomy along with resection of the primary tumor. Of this group, 45 patients had complete response of metastases on review of final pathology.^79^ This group was found to have significantly lower blood CEA levels. Such findings suggest that future studies should investigate the role of elevated CEA as a negative prognostic factor when deciding whether or not to perform metastasectomy.

### Inflammatory Markers

Recently, the concept of systemic inflammation in carcinogenesis has been leveraged in an attempt to identify markers of disease and treatment response. A study by Kim et al. investigated whether inflammatory markers could serve as prognostic indicators in patients with advanced PDAC undergoing gemcitabine-based chemotherapy. They found that neutrophil-to-lymphocyte ratio (NLR), platelet to lymphocyte ratio (PLR), and C-reactive protein to albumin ratio were independent predictors of overall survival.^80^ Despite such efforts, no individual level or ratio has been found to be sufficiently sensitive and specific, and therefore, several scoring systems have been proposed.

A study by Nurmi et al. combined CRP and CA19-9 into a prognostic score and examined patients with resectable or borderline-resectable PDAC. They found that patients with CRP and CA19-9 below a cutoff value (3 mg/l and 3700 U/L, respectively) had a disease-specific survival time of 54 months compared with only 16 months in patients whose CRP and CA19-9 were above the cutoff values.^81^

Other scoring tools have been suggested. A modified Glasgow prognostic score has been proposed that incorporates albumin and CRP levels as prognostic markers.^82^ The systemic inflammatory index (SII) was found to be an independent negative predictor of overall survival and is calculated by multiplying neutrophil and monocyte counts, then dividing them by the lymphocyte count.^83^ Frigerio et al. found that, in patients who had liver-only synchronous metastases and underwent pancreatectomy after complete regression of the metastases, NLR and SII were associated with overall survival.^46^

While several of these inflammatory markers show some promise, data have been inconsistent, and large-scale studies, particularly in oligometastatic disease, are warranted to define their role in guiding treatment decisions.

### Liquid Biopsy Modalities

Liquid biopsy has been increasingly utilized in CRC, breast, and lung cancers to monitor treatment efficacy, disease progression, and therapy resistance.^84^ Various methods of liquid biopsy have been described, including circulating tumor cells (CTCs), cell-free nucleic acid (cfDNA and cfRNA), and extracellular vesicles, such as exosomes.

Circulating tumor DNA (ctDNA) is a small subpopulation derived from cfDNA. A recent meta-analysis of liquid biopsy methods found that the use of ctDNA in the diagnosis of PDAC had a sensitivity of only 0.64.^85^ Early in PDAC progression, the rate of necrosis and apoptosis is low, and only one ctDNA molecule may be detected in 5 ml of plasma, which could account for this result. However, ctDNA may have a more substantial role in the detection and monitoring of advanced PDAC. In a study of patients with resectable localized PDAC, ctDNA detection in the preoperative setting was associated with poorer recurrence-free survival and overall survival.^86^ Uesato et al. evaluated PDAC patients with liver metastasis and found that patients with detectable ctDNA levels had worse overall survival. The presence of ctDNA also significantly correlated with a higher number of liver metastases, lung and/or peritoneal metastases, and higher Ca19-9 levels.^87^

Beyond just quantitative measurements of cfDNA, assessment of the mutational landscape also might be illuminating. Several studies have shown that levels of KRAS mutation in cfDNA correlated with radiographic tumor response to therapy in patients with mPDAC and predicted early recurrence following curative-intent resection.^88^ A recent study of 512 patients with PDAC found that ctDNA KRAS mutations were detected in 57% of patients, and the frequency of KRAS mutation differed depending on the metastatic organ. The KRAS mutation detection rate was significantly higher in patients with metastasis to the liver (78%) compared with lung (46%) and lymph nodes (60%).^89^ Although the sensitivity and specificity of such assays is still lacking, identification of such genetic mutations may reveal biologic subtypes that can help to guide operative decision-making. Further studies in oligometastatic PDAC should explore the impact of such mutations on prognosis and tumor behavior.^90^

Circulating tumor cells (CTCs) also have been studied in a variety of cancers, and in some cases their levels may indicate distant disease.^91^ Court et al. found that PDAC patients with occult metastatic disease had significantly more CTCs measured preoperatively compared with patients who had local disease only. They also found CTCs to be an independent predictor of recurrence-free survival after surgery.^92^

CLUSTER, a prospective longitudinal study, found that patients who received preoperative chemotherapy had significantly lower CTCs, and surgical resection of the tumor resulted in significant reduction of CTCs. Preoperative numbers of CTCs also were found to be predictors of early recurrence within 12 months from surgery.^93^

As we learn more about liquid biopsy techniques, findings should be correlated with current clinical practices (such as tumor markers and radiographic markers) to determine their clinical utility in following cancer progression and recurrence. Clarification on the role of liquid biopsy in PDAC management will create more datapoints for operative decision-making.

## Conclusions

Metastasectomy for PDAC has increased in recent years, despite a lack of consensus definition for what constitutes oligometastatic disease. The majority of studies have focused on liver and lung metastases, with some exploration into peritoneal disease. The most common organ for metastatic spread and distant recurrence—the liver—also is the site associated with the worst prognosis compared with other sites of metastatic spread. In this population, data suggest that incorporating systemic chemotherapy before operative resection is associated with improved survival, emphasizing the importance of attempting to “downstage” the tumor. In patients with pulmonary metastases, several studies have reported longer overall survival times in patients with longer disease-free intervals.

Most of the data available are retrospective in nature, with significant variability in methodology and results reporting. Often, these investigations have been done in experienced academic centers, making the results difficult to generalize. More importantly, it is worth noting that there is a selection bias inherent to these studies; patients undergoing metastasectomy with subsequently increased overall survival likely had favorable tumor biology to begin with. Our review of the data shows that response to chemotherapy, normalization of biochemical markers, and longer disease-free intervals are associated with improved overall survival. This demonstrates that selection bias is not a flaw; it reveals a crucial detail about attempts to predict tumor behavior.

Current methods for estimating tumor biology are rudimentary, with varying levels of accuracy. At present, the best proxy for PDAC behavior is a response to chemotherapy, measured by some combination of trends in biochemical markers (such as Ca19-9) and radiographic findings or the rate of tumor growth and/or development of metastases. We now have under investigation newer modalities, such as liquid biopsy techniques, and investigations of the mutational landscape, which, although in their early stages, have shown promising results. As we gain a better understanding of the complexity of PDAC on a cellular and molecular level, we must move towards a personalized approach when selecting therapies. Investigating these individualized methods of predicting biologic behavior will enable a better understanding of those patients who would benefit from aggressive surgical approaches and metastasectomy.
